# Analysis of licensed South African diagnostic imaging equipment

**DOI:** 10.11604/pamj.2015.22.57.7016

**Published:** 2015-09-18

**Authors:** Joseph Mwamba Kabongo, Susan Nel, Richard Denys Pitcher

**Affiliations:** 1 Division of Radiodiagnosis, Department of Medical Imaging and Clinical Oncology, Faculty of Medicine and Health Sciences, Stellenbosch University, Tygerberg, South Africa; 2 Department of Health Directorate, Radiation control, Bellville, South Africa

**Keywords:** Diagnostic imaging equipment, resource-limited environment, national health insurance

## Abstract

**Introduction: Objective:**

To conduct an analysis of all registered South Africa (SA) diagnostic radiology equipment, assess the number of equipment units per capita by imaging modality, and compare SA figures with published international data, in preparation for the introduction of national health insurance (NHI) in SA.

**Methods:**

The SA Radiation Control Board's database of registered diagnostic radiology equipment was analysed by modality, province and healthcare sector. Access to services was reflected as number of units/million population, and compared with published international data.

**Results:**

General X-ray units are the most equitably distributed and accessible resource (34.8/million). For fluoroscopy (6.6/million), mammography (4.96/million), computed tomography (5.0/million) and magnetic resonance imaging (2.9/million), there are at least 10-fold discrepancies between the least and best resourced provinces. Although SA's overall imaging capacity is well above that of other countries in sub-Saharan Africa, it is lower than that of all Organisation for Economic Co-operation and Development (OECD). While SA's radiological resources most closely approximate those of the United Kingdom, they are substantially lower than the UK.

**Conclusion:**

SA access to radiological services is lower than that of any OECD country. For the NHI to achieve equitable access to diagnostic imaging for all citizens, SA will need a more homogeneous distribution of specialised radiological resources and customized imaging guidelines.

## Introduction

Globally, governments are experiencing increasing pressure to fund essential public-sector services [1]. This is particularly true for healthcare, where worldwide expenditure currently exceeds $4 trillion, representing 9 per cent of the global Gross Domestic Product (GDP) [2]. However, there is wide discrepancy in international healthcare spending, with amounts ranging from less than $3 per person annually in some low-income African countries, to $6,250 per person (18% of GDP) in the United States of America (USA). In more than 30 countries, the annual per capita healthcare expenditure is less than $20 [1]. Thus, while many low-income countries have a high burden of disease, they lack healthcare infrastructure [2]. This is particularly true in sub-Saharan Africa (SSA), which is home to 11 per cent of the world's population and bears 24 per cent of the global disease burden, but has only 3 per cent of the world's health workers and accounts for less than one per cent of global healthcare spending [2]. Like other SSA countries, South Africa (SA) faces substantial healthcare challenges, including dual HIV and PTB pandemics, a high infant mortality rate, and an increasing burden of non-communicable diseases and trauma [3, 4]. SA has a population of almost 53 million people and currently spends approximately 8.5 per cent of GDP on healthcare, which is in line with levels observed in Organisation for Economic Co-operation and Development (OECD) countries. Approximately 17 per cent of the SA population (9 million people) has access to private healthcare; the remaining 83% (44 million people) is dependent on public-sector resources. However, the private sector accounts for approximately half of all SA healthcare expenditure (5.1% of GDP) and employs 70% of healthcare specialists, thereby providing a well-developed private-sector infrastructure; conversely, the public sector is relatively under-resourced [5]. It has been shown that public-private partnerships can enhance the quality of existing secondary and tertiary services while extending the provision of primary care [1]. Many governments are thus turning to the private sector in an attempt to address public healthcare needs. It is in this context that the SA Government will be introducing National Health Insurance (NHI) [6]. There is a burgeoning global demand for diagnostic imaging [7–9] and over the past three decades, basic radiological services have increasingly been viewed as an essential component of healthcare [10–12]. Furthermore, although radiological equipment is expensive and imaging constitutes a significant proportion of healthcare expenditure [13], the value added by radiological services to both the individual patient and the sustainability of healthcare systems has been increasingly acknowledged [14]. Ensuring equitable access to radiological services is thus pivotal to the successful implementation of any healthcare system. South Africa's National Health Insurance system will be no different. Accurate knowledge of SA's diagnostic imaging capacity is therefore important. Although the Radiation Control Board maintains an accurate database of licensed diagnostic imaging equipment in SA, to the best of our knowledge, there has been no unifying analysis of these data, to assess South Africa's overall per capita imaging capacity by diagnostic modality. Furthermore, to the best our knowledge, there has been no comprehensive analysis of the diagnostic imaging capacity of any country in sub-Saharan Africa. The aim of this study is to: i. Conduct an analysis of all registered diagnostic radiology equipment in South Africa ii. Assess the number of equipment units per capita by imaging modality, iii. Compare SA figures with available published international data.

## Methods

The database of the SA Radiation Control Board; which includes an inventory of all licensed SA diagnostic radiology units including general or conventional radiography (GR), fluoroscopy (FL), mammography (MM), digital subtraction angiography (DSA), computerized tomography (CT), magnetic resonance imaging (MRI) and positron emission tomography/computed tomography (PET/CT) equipment, was captured on an MS Excel spread sheet and analysed by modality, province and healthcare sector. All general purpose, fixed radiology installations were included. Mobile, industrial and mass screening units were excluded. Amongst radioisotope equipment, only positron emission tomography/computer tomography (PET/CT) was considered. For each imaging modality, access to radiological services in the public, private and combined sectors was reflected as the number of units per million population, based on the 2013 South African mid-year population estimates [15]. The public sector refers to government medical institutions and the private sector to any other medical facility with registered diagnostic radiology equipment (private radiologist, other medical specialist, general practitioner, chiropractor, private radiographer, mining company and private medical institution). It was assumed that 17% of the SA population has access to private medical services. The number of SA units per million population for each imaging modality was then compared with available published OECD data, thereby providing a comparative assessment of South Africa's capacity to deliver a national diagnostic imaging service. Although the WHO has published estimated data on the diagnostic imaging capacity of all countries, figures are based on national surveys and interviews, rather than national equipment databases and therefore were excluded from comparison.

**Institutional board review approval**: Stellenbosch University Health Research Ethics Committee reference S14/02/030.

## Results

**Figure 1:**
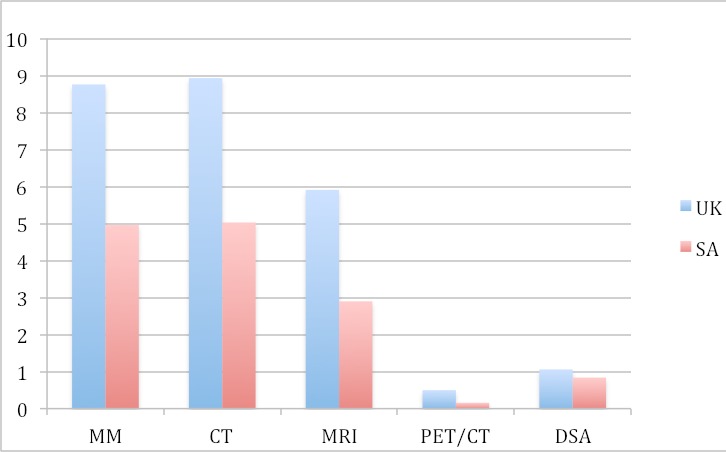
A comparison of SA and UK data by imaging units per million population

South Africa's diagnostic imaging equipment resources are reflected in Table 1. Only three of SA's eleven provinces (Gauteng, Western Cape Province and Kwazulu-Natal) have the full spectrum of diagnostic imaging modalities in both the public and private sectors. Overall, Gauteng has the best provincial resources and Mpumalanga the least. General radiography is the most accessible modality, with 34.8 units per million population. Geographically, this is also the most equitably distributed modality, with overall the smallest discrepancy in units per million between the least and best-resourced provinces (1:2.3), and between the public and private sectors (1: 5.3). Although the country's overall number of units per million population is similar for fluoroscopy (6.6), mammography (4.96) and CT (5.0), these modalities show an average 11-fold discrepancy between the least and best resourced provinces and an average 13-fold discrepancy between the public and private sectors. Currently, the Northern Cape has no public sector mammography service. The 2.9 MRI units per million population largely reflect private sector capacity, with a 46-fold overall discrepancy in MRI resources between the public and private sectors. Mpumalanga and the North West Province have no public sector MRI service. Table 2 compares South Africa's diagnostic imaging capacity with published international data [16, 17]. Of note, there are no OECD data for general radiography or fluoroscopy. Furthermore, there are currently no comprehensive comparative data on imaging capacity in other African countries. For every modality, South Africa's overall imaging capacity is lower than any OECD country. While SA resources most closely approximate those of the United Kingdom (UK), they are nonetheless substantially lower than the UK (Figure 1).

**Table 1:** South Africa diagnostic radiology resources by modality per million population

Province (Population)	General radiography				Fluoroscopy			Mammography			Computed tomography			Magnetic Resonance			Digital Subtraction Angiogram			Positron Emission Tomography/Computer Tomography		
	Total		Public	Private	Total	Public	Private	Total	Public	Private	Total	Public	Private	Total	Public	Private	Total	Public	Private	Total	Public	Private
**Eastern Cape (6620100)**	26.1		18.7	111.4	2.9	1.31	20.7	3.7	2.4	18.8	2.8	1.3	20.7	1	0.1	11.3	0.45	0.32	1.88	0	0	0
**Free State (2753200)**		41.8	27.5	**312.4**	6.2	1.9	**87.1**	6.1	**2.6**	**72.6**	6.5	**3.4**	**65.3**	2.5	0.3	2.5	1.08	**1.14**	0	0	0	0
**Gauteng (12728400)**		41.3	16.4	87.8	**13.8**	**5.2**	29.8	**9.3**	1.8	23.3	**8.8**	2.2	21.1	4.8	0.3	4.8	**1.72**	0.84	**3.36**	**0.39**	**0.24**	0.67
**Kwazulu-Natal (10456900)**		29.4	18.8	84.9	6.3	2.8	17.3	3.4	0.7	17.3	4.2	1.7	17.3	2	0.5	2	1.05	0.79	2.39	0.19	0.11	0.59
**Limpopo (5518000)**		29.2	20.0	203.0	2.2	1.1	21.7	1.2	0.3	18.1	1.1	1.1	14.4	0.7	0.1	0.7	0.36	0.38	0	0	0	0
**Mpumalanga (4128000)**		29.2	16.6	266.5	1.5	1.0	6.9	0.7	0.5	3.4	0.7	0.5	3.4	1.9	0	1.9	0	0	0	0	0	0
**Northern Cape (1162900)**		**61.1**	**43.9**	90.2	5.2	2.6	12.8	1.7	0	8.5	3.4	1.7	8.5	1.7	**0.8**	1.7	0	0	0	0	0	0
**North West (3597600)**		31.4	16.6	50.1	3.0	0.6	25	2.2	0.5	16.6	2.2	0.5	16.6	0.8	0	0.8	0	0	0	0	0	0
**Western Cape (6016900)**		39.2	24.5	116.3	9.3	3.2	41.5	7.6	1.5	39.4	7.9	2.7	35.3	**6.6**	0.5	**38.4**	0.66	0.39	2.07	0.33	0.19	**1.03**
**Total (52982000)**		34.8	19.8	104.0	6.6	2.5	26.8	4.96	1.29	22.3	5.0	1.7	20.7	2.9	0.3	15.14	0.84	0.51	2.63	0.16	0.08	0.59
**Last: Best**		2.3	2.6	6.2	9.2	8.6	12.6	1.3	N/A	21.3	12.5	6.8	19.2	9.4	N/A	54.8	N/A	N/A	N/A	N/A	N/A	N/A
**Private: Public**	5.3				10.7			17.3			12.1			45.8			5.15			7.37		

South Africa's Country Profile: Population 52 982 000 with General Radiography 34.8, Fluoroscopy 6.6, Mammography 4.9, Computer Tomography 5, Magnetic Resonance Imaging 2.9, Digital Subtraction Angiography 0.8 and Positron Emission Tomography 0.2 units per million population. Only three provinces (Gauteng, Western Cape and Kwazulu-Natal) have the full spectrum of diagnostic imaging modalities in both the public and private sectors. Overall, Gauteng has the best provincial resources and Mpumalanga the least

**Table 2:** Published international diagnostic imaging equipment resources by modality per million population - OECD/AFRICA

MM	per million	CT	per million	MRI	per million	PET/CT	per million	DSA	per million
Korea	54.82	Japan	101.28	Japan	46.87	United states	5	Switzerland	27.78
United States	40.17	United states	40.89	United states	34.45	Switzerland	3.39	Finland	19.58
Switzerland	33.06	Iceland	40.68	Korea	23.46	Korea	3.82	Australia	16.58
Japan	31.58	Korea	37.08	**OECD Average**	**23.2**	Japan	3.65	Iceland	15.65
Finland	31.22	Switzerland	34.82	Iceland	21.9	Finland	2.22	**OECD Average**	**11.22**
**OECD Average**	**26.46**	Finland	21.8	Finland	21.61	**OECD Average**	**2.21**	United states	10.37
New Zealand	24.81	**SA private**	**20.7**	**SA private**	**15.14**	Australia	1.85	Korea	7.96
Australia	22.79	Ireland	17.34	Australia	15.03	Ireland	1.8	New Zealand	4.96
**SA private**	**22.33**	New Zealand	15.34	Ireland	12.83	France	1.36	Chile	3.96
Iceland	15.65	Canada	14.62	New Zealand	11.05	Canada	1.2	Israel	3.68
Ireland	14.18	France	13.52	Canada	8.83	New Zealand	1.13	**SA private**	**2.63**
Chile	14.08	**OECD Average**	**13.3**	Slovenia	8.75	**SA private**	**0.59**	**United Kingdom**	**1.06**
**United Kingdom**	**8.76**	Chile	11.21	France	8.66	**United Kingdom**	**0.5**	**South Africa**	**0.84**
**South Africa**	**4.96**	Israel	9.26	**United Kingdom**	**5.91**	Chile	0.4	**SA public**	**0.51**
**SA public**	**1.29**	**United Kingdom**	**8.92**	Chile	4.42	**South Africa**	**0.16**		
Kenya	0.5	**South Africa**	**5.03**	Israel	2.92	**SA public**	**0.08**		
Ghana	0.3	**SA public**	**1.71**	**South Africa**	**2.9**				
Uganda	0.2	Kenya	0.8	Egypt	2				
		Ghana	0.5	**SA public**	**0.33**				
		Uganda	0.3						

OECD: Organisation of Economic Cooperation and Development, MM: Mammography, CT: Computer Tomography, MRI: Magnetic Resonance Imaging, PET/CT: Positron Emission Tomography/Computer Tomography, DSA: Digital Subtraction Angiography, SA: South Africa.

## Discussion

This study is the first comprehensive analysis of the diagnostic imaging capacity of an African country and as such adds important new insights into the provision of healthcare on the African continent. Furthermore, our analysis has revealed that SA's overall diagnostic imaging capacity most closely approximates that of the United Kingdom, while being substantially less than the UK. This is a cardinal observation, since the UK has an existing National Health Service (NHS) and also has published national imaging protocols, as promulgated by the Royal College of Radiologists (RCR) [18]. The imaging model of the United Kingdom could thus potentially serve as a model for development of imaging capacity, and the evolution of referral patterns, in a unified South African healthcare system. During the course of this research project, and prior to the publication of our audit findings, the Radiological Society of South Africa (RSSA) fortuitously adopted the RCR referral guidelines, with minor modifications, with a view to implementation in South Africa, in both the private and public health sectors [19]. This serendipitous development makes our analysis particularly relevant to healthcare in South Africa. Based on our findings, cautious implementation of the RCR guidelines would certainly appear to be appropriate in the South African context. Reference to Table 2 reveals that the diagnostic imaging capacity within the SA private sector is superior to that in the UK for all modalities. This suggests that the RCR referral guidelines can be relatively easily accommodated within the South African private sector. However, resources in the SA public sector are substantially lower than those in the UK. The impact of the RCR referral protocols on the SA public sector will have to be carefully monitored, and guidelines will have to be modified as required, to ensure that the proposed referral patterns are sustainable within the public sector. This is particularly true if one considers the countries’ respective disease profiles (Table 3). South Africa has a substantially higher burden of HIV, TB and trauma, while the United Kingdom, has a significantly larger aging population. In addition, a major SA challenge will be to address the geographical discrepancies in distribution of imaging equipment identified in this study. If SA is to achieve equitable access to diagnostic imaging, the least-resourced provinces will have to be afforded priority when allocating future equipment resources. A constraint to achieving OECD-level imaging capacity in SA is the discrepancy in GDP between the average OECD country and SA. The OECD average total expenditure on health is approximately 9.4% of GDP, which represents $3701 per capita, while SA's 8.5% of GDP spent on healthcare represents just $645. Unifying the private and public sector imaging platforms under the proposed NHI, would appear to be an important first step in achieving more equitable access to imaging for the South African population. However, to ensure long-term viability, referral protocols would have to be constantly monitored and refined to yield customized imaging protocols appropriate to a resource-limited environment.

**Table 3:** Comparative data of South Africa-United Kingdom-OECD average

OECD FACT BOOK 2014/WORLD BANK 2011 PUBLISHED IN 2013	SOUTH AFRICA			UNITED KINGDOM			OECD Average		
Expenditure on Health	**PU**	**PR**	**TOTAL**	**PU**	**PR**	**TOTAL**	**PU**	**PR**	**TOTAL**
	3.5	5.1	**8.5**	7.8	1.6	**9.4**	6.7	2.6	**9.4**
Population	52 396 000			63 705 000			1 205 407 000		
Population Growth Rate%	0.84			0.66			0.66		
Working Population%	65			65.4			66.6		
Elderly%	5			17			15		
GDP per Capital-$	645			3647			3701		
Real GDP Growth (2012)%	2.5			0.1			1.5		
CPI Inflation (2012)%	14.6			4.9			4.1		
Disease: TB/100000	1003			15			X		
Trauma	59935			X			X		
Maternal Health Mortality/100 000	140			8			X		
Health Care System	No NHS: Public and private			NHS			NHS		

OECD: Organisation for Economic Cooperation and Development, PU: public, PR: private, GDP: Gross Domestic Product per capital-Health expenditure, CPI: Consumer Price Index, TB: Tuberculosis, HIV: Human Immunodeficiency Virus, NHS: National Health System

## Conclusion

This analysis demonstrates an inhomogenous provincial distribution of radiological equipment, with a wide discrepancy between the public and private health sectors, and lower overall access to radiological services than any OECD country.
